# Editorial: Lessons from external quality control in laboratory medicine: important implications for public health!

**DOI:** 10.3389/fmolb.2024.1485193

**Published:** 2024-09-12

**Authors:** Nathalie Weiss, Ingo Schellenberg, Klaus-Peter Hunfeld

**Affiliations:** ^1^ INSTAND e.V., Society for Promoting Quality Assurance in Medical Laboratories e.V., Duesseldorf, Germany; ^2^ Center of Life Sciences, Institute of Bioanalytical Sciences (IBAS), Anhalt University of Applied Sciences, Bernburg, Germany; ^3^ Northwest Medical Centre, Academic Teaching Hospital, Medical Faculty, Institute for Laboratory Medicine, Microbiology & Infection Control, Goethe University Frankfurt, Frankfurt, Germany

**Keywords:** proficiency testing (PT), external quality assessment (EQA), public health, laboratory medicine, longitudinal analysis

## 1 Introduction

Laboratory medicine has gone a long way from the optical and olfactory analysis of urine in the 16^th^ century to modern day laboratory diagnostics, where even a small amount of nucleic acid from human pathogens can be detected out of a matrix of a variety of other molecules. Moreover, currently, up to 80% of medical decision making is supported by laboratory analysis, highlighting the substantial impact of laboratory medicine on public health (Salinas, 2023). The need for reliable laboratory results, regardless of testing site or method, gained international attention when a survey by Belk and Sunderman showed that 42% of laboratory results for glucose and 49% for hemoglobin were of insufficient quality (Belk et al., 1947). Then, as now, those figures were unacceptable and posed a clear threat to public health. This publication is often cited as the kick-off for the success story of external quality assessment (EQA) schemes, also known as proficiency testing (Doxiadis et al., 2024). Today, EQA schemes are a mandatory part of various national and international guidelines like the Clinical Laboratory Improvement Amendments of 1988 (CLIA) of the United States of America (USA) (Clinical laboratory improvement Amendments of 1988) or the Guideline of the German Medical Association (Richtlinie der Bundesärztekammer Rili-BÄK) (Bundesärztekammer, 2023). In addition, laboratories seeking accreditation, e.g., according to ISO 15189, are obliged to participate in external quality assurance procedures (ISO, 2023).

The organization of EQA schemes is mostly done by non-profit organizations like INSTAND e.V., CAP or UK-NEQAS and some commercial institutions like BioRad and all institutions are accredited according to ISO 17043. Although the guideline provides a good framework for the organization of high-quality EQA schemes, EQA providers still face some hurdles such as the provision of suitable samples for the quality assessment. One intensely discussed Research Topic is the commutability of EQA samples, meaning their exchangeability with patient samples. In their opinion paper, Vierbaum et al. address the currently published models of commutability assessment and discuss them in the context of feasibility particularly for the providers of EQA schemes.

This feasibility is especially challenged when new analytes or methods are introduced to the *in vitro* diagnostic market. A growing market is focused on point-of-care testing (POCT, also known as bedside diagnostics) and the analytical increase in this area of laboratory medicine has also been observed by Luppa et al. for the detection of glucose with a rise in participating laboratories in the POCT glucose EQA program in Germany. In addition, they put the EQA results for HbA1c and POCT-glucose in perspective to the current diagnostic methodology of tests for diabetes as well as morbidity and mortality of diabetes patients, showing that the quality of the measurement of HbA1c clearly improved over time.

The positive impact of accreditation status and analytical methods on the likelihood of satisfactory results in the detection of *Escherichia coli* in environmental samples by Canadian environmental testing laboratories was demonstrated by Sreya et al. and the authors propose an implementation of regulated EQA schemes in drinking water safety plans.

New clinical variants of bacteria challenge laboratories and physicians alike, and therefore it is important to include these variants in quality assessments for training and for testing the quality of the assays used. In support, Kremser et al. showed a positive effect of using new clinical variants for EHEC/STEC, *B. burgdorferi* and MRSA/cMRSA in their longitudinal evaluation of EQA schemes for the detection of these bacteria using nucleic acid amplification techniques (NAAT).

Bacteria pose a growing threat to public health due to increasing antimicrobial resistance (AMR) (Antimicrobial Resistance Collaborators, 2022; The Lancet, 2024). Therefore, the correct identification and subsequent susceptibility testing of bacteria is of paramount importance for therapy and care of infectious diseases. Here, Lindenberg et al. examined 17 years of EQA schemes for bacterial identification and susceptibility testing and showed that while the quality of bacterial identification remained consistently high, the quality of AMR testing was affected by laboratory type as well as changes in testing guidelines and unregulated adherence to these guidelines.

The recent COVID-19 pandemic and the Mpox outbreaks have brought the detection of viral genetic material by NAAT into the focus of specialists and manufacturers. In particular, new NAAT methods for whole genome (WG) sequencing and the subsequent encouragement by authorities to use this method have raised questions about the current quality of these methods. Interestingly, Camp et al. identified hurdles in building next-generation sequencing capacity in diagnostic laboratories in Austria, but the overall quality of analyses was good, with a few exceptions that clearly showed improvement in quality over time.

While the pandemic shifted the focus from classical laboratory medicine more to the detection of infectious diseases, other analytes gained also in importance. Accordingly, Kirschfink et al. observed an increasing interest in complement EQA programs since 2016. While the pass rates for C3, C4, C1 inhibitor antigen and activity determinations provided good proficiency testing results, the activation pathways showed greater variance, especially for pathological samples, highlighting the need for further improvement and harmonization.

However, there is also room for improvement for well-established biomarkers such as high-sensitivity (hs)-CRP. Weiss et al. were able to show that there is a positive trend towards harmonization, based on EQA data. However, the persistence of manufacturer-specific differences underlines the further need for meta-analytics stratified by assay in order to gain a meaningful insight into the usefulness of this marker for cardiovascular risk assessment. Similarly, the analyses by Toll et al. show the interesting evolution of the EPO EQA program, that was first introduced in 2017, from its first steps till now. It highlights the difficulties and opportunities of new EQA surveys for blood and serum markers and the potential impact of reference materials and methods is discussed further. Kremser et al. also emphasized the need for international reference materials based on their longitudinal evaluation of EQA schemes for cancer antigen tumor markers. The methods used by the different laboratories showed high precision within methods but considerable variability between methods, underlining the fact that the same patient should only be monitored with the same method.

For EQAs the meaningful analysis and interpretation of statistical data is extremely important. While most EQA programs can only be evaluated based on the consensus mean or results from expert laboratories due to the lack of a reference method and reference material, these are established for some markers such as steroid hormones. In their study, Vierbaum et al. were able to determine the accuracy of several immunoassays for the detection of testosterone, progesterone and 17β-estradiol in serum based on EQA data. Whereas improvement in standardization is required for accurate analysis and thus clinically reliable interpretations, one manufacturer showed increasing accuracy over the observed time period.

Unfortunately, the statistical analysis of analytes in biological matrices such as blood or urine is not as simple, as the values are not guaranteed to follow a normal distribution. Seifert et al. propose a logit transformation for the analysis of data points near 0% or 100% to generate a symmetric distribution with zero center, so that parametric statistical methods can be used without bias.

And while generating reliable laboratory results is important for medical diagnosis, one important factor should never be overlooked: The patient is more than just a measurable analyte. In a medico philosophical article Reiber’s hypothesis and theories are discussed to promote the need and opportunity for CSF diagnostic reports that integrate all patient data rather than looking at individual markers, which serves better care for the individual patient but can also help reducing costs for the healthcare system as a whole.

## 2 Conclusion and perspective

Overall, this Research Topic on external quality control in laboratory medicine provides an excellent overview of the impact and importance of proficiency testing in different areas of laboratory diagnostics and public health. The different authors from various areas of laboratory medicine have not only provided a well-rounded picture of proficiency testing tools and the impact of the obtained results on public health and quality of care but, most importantly, have also shared their critical thoughts and opinions on current principles and future opportunities for EQA in the years to come.
